# Revisiting the Ross operation: early results from a new Ross program at a cardiovascular center in South America

**DOI:** 10.3389/fcvm.2026.1837210

**Published:** 2026-06-15

**Authors:** Santiago Besa, Álvaro Torres, Pedro Ugarte, Cecilia Romero, Rodrigo González, Pedro Becker, Günther Krögh

**Affiliations:** 1 Sección de Cirugía Cardíaca, Escuela de Medicina, Pontificia Universidad Católica de Chile, Santiago, Chile; 2 Red de Salud UC CHRISTUS, Santiago, Chile; 3 Escuela de Medicina, Pontificia Universidad Católica de Chile, Santiago, Chile

**Keywords:** aortic valve surgery, Latin America, postoperative complications, programmatic implementation, pulmonary autograft, Ross procedure

## Abstract

**Introduction:**

Aortic valve disease in young adults remains challenging due to long life expectancy and the need for durable, anticoagulation-free solutions. The Ross procedure offers excellent hemodynamics and long-term durability but is underutilized in Latin America because of its technical complexity and limited homograft availability.

**Methods:**

We conducted an observational study including all adult patients undergoing the Ross procedure between July 2024 and April 2026 at Hospital Clínico UC-Christus. Demographics, clinical history, surgical indications, operative details, complications, and echocardiographic follow-up were analyzed.

**Results:**

29 consecutive patients were included (13.8% female; range: 21–61). Main surgical indications were severe aortic stenosis (34.5%), mixed lesions (34.5%) and severe aortic regurgitation (27.6%); 86.2% had bicuspid aortic valves. Associated procedures were performed in 24.1% of cases. Median cross-clamp and cardiopulmonary bypass times were 205 and 230 min, respectively. Early complications included perioperative stroke in 3 patients (10.3%; all ischemic, two recovered with mRS: 0–1 and one with mRS: 3). Early echocardiography follow-up showed no neoaortic regurgitation in 18 patients, trivial in 9, and mild in 2, with a median neoaortic gradient of 4.0 mmHg and neopulmonary gradient of 2.0 mmHg. No patient developed severe autograft or homograft regurgitation. During a median follow-up of 295 days (range: 30–662), no deaths were observed. There was 1 reintervention due to homograft endocarditis.

**Conclusion:**

Implementation of a dedicated Ross program in a high-complexity Latin American center is feasible and was associated with acceptable early postoperative outcomes. These findings support feasibility and short-term safety only; longer follow-up and larger cohorts are required before conclusions can be drawn regarding durability or wider safety.

## Introduction

Aortic valve replacement in young and middle-aged patients remains a major clinical challenge due to their long-life expectancy and high functional demands. Although mechanical and bioprosthetic valves reliably relieve left ventricular outflow obstruction, they do not reproduce the native aortic root's biological and hemodynamic properties, exposing patients to either lifelong anticoagulation or structural valve degeneration. In contrast, the Ross procedure, which consists of replacement of the diseased aortic valve with the patient's own pulmonary autograft and reconstruction of the right ventricular outflow tract, provides a living valve substitute capable of growth, remodeling, and adaptation to systemic hemodynamic conditions, with near-normal transvalvular gradients and ventricular loading (1–3). Moreover, survival after the Ross operation parallels that of the age- and sex-matched general population and exceeds that observed with both mechanical and bioprosthetic valves (4).

Despite the growing body of evidence supporting the Ross procedure, its wide adoption has been limited. This is driven, at least in part, by the technical complexity of the operation, concerns about long-term autograft and homograft durability and homograft availability (1, 2, 5).

Historically, in our institution the Ross procedure was reserved for the pediatric population and for young women of childbearing age, reflecting longstanding concerns regarding its long-term durability and the position of mechanical aortic valve replacement as the gold-standard intervention for most adults (6). However, the emerging contemporary evidence demonstrating improved outcomes led us to reconsider its role. Consequently, we have expanded its indication and initiated a dedicated adult Ross program. The present study therefore describes the implementation of an institutional Ross surgery program in a high-complexity university hospital in Latin American context, detailing patient selection, operative strategy, and early clinical and hemodynamic outcomes as a foundation for future regional practice and benchmarking.

## Patients and methods

We conducted a single-center, observational and descriptive study including all consecutive adult patients who underwent the Ross procedure from the start of the program in July 2024 through April 2026 at the Department of Cardiovascular Surgery, Pontificia Universidad Católica de Chile. Eligibility was assessed during a weekly multidisciplinary Heart Team conference and discussed individually with the patient, and mainly took into account criteria proposed by Abolenazar and colleagues (7) and is summarized in Table 1 (age < 60 years, absence of connective-tissue disorder, life expectancy > 15 years), together with individual anatomical characteristics, activity profile, and the patient's preference for an anticoagulation-free solution. During the study period, 158 adult patients underwent isolated or combined aortic valve surgery at our institution; of these, 29 (18.3%) underwent the Ross procedure and represent the cohort analyzed in the present study. Two attending cardiac surgeons with dedicated training in the Ross operation (SB, GK) performed all cases, always assisted by a consistent perfusion, anesthesia, and nursing team, which was considered a prerequisite for program initiation.

**Table 1:** Institutional criteria for patient selection and technical considerations for the Ross procedure.

Category	Clinical or anatomical characteristics	Comments/technical considerations
Ideal candidates	- Young or middle-aged adults (<60 years) with non-repairable aortic valve disease. - Predominantly severe **aortic stenosis**. - Normal or small aortic annulus (<27 mm). - Normal ascending aortic diameter (<40 mm).	Expected to achieve long-term durability and survival comparable to the general population. Best results in centers with dedicated Ross programs.
Favorable clinical profiles	- **Active lifestyle** or athletic patients. - **Women of childbearing potential** seeking to avoid anticoagulation. - Patients with contraindications or unwillingness to receive lifelong warfarin.	Avoidance of anticoagulation and superior hemodynamics make the Ross procedure particularly advantageous in these subgroups.
Acceptable with modified techniques	- **Aortic regurgitation** or mixed lesions (stenosis + regurgitation) with stable root anatomy. - **Dilated annulus (≥27 mm):** may undergo external annuloplasty (PTFE or Dacron ring). - **Ascending aorta 38–40 mm:** consider short Dacron interposition graft to stabilize sinotubular junction.	Requires technical modifications: deep sub-annular full-root replacement combined with external annuloplasty ring and/or synthetic graft reinforcement of the sinotubular junction or ascending aorta. A Dacron-encased inclusion strategy has NOT been adopted in our program.
Anatomical variants permitting surgery	- **Bicuspid aortic valve (BAV):** acceptable **if no associated aortopathy** or connective tissue disease. - **Endocarditis:** indicated when avoidance of prosthetic material is desirable and infection is localized.	BAV does not increase autograft dilatation risk in absence of connective tissue disorder. The Ross operation provides good infection control in endocarditis cases.
Contraindications	- **Connective tissue disorders** (e.g., Marfan, Loeys-Dietz, Ehlers-Danlos). - **Familial or syndromic aortopathy.** - **Advanced comorbidities** limiting life expectancy (<15 years). - **Active systemic autoimmune disease** (e.g., lupus, rheumatoid arthritis).	High risk of autograft dilatation and early failure; other valve substitutes are preferred.

Data were collected from institutional medical records, surgical databases, and outpatient follow-up visits. Extracted variables included demographic characteristics, relevant clinical history, surgical indication, operative technique, intraoperative and postoperative complications, and echocardiographic follow-up parameters. The results are described as absolute and relative frequencies for categorical variables and measures of central tendency and dispersion for continuous variables. Data were processed, analyzed, and summarized using R (version 4.5.0) through RStudio (version 2025.05.0+496; Posit Software, PBC).

All surgeries were performed under mild hypothermia (32–34 °C) using exclusively anterograde Del Nido cardioplegia. In our program, the pulmonary autograft is implanted as a free standing full-root replacement as described by El-Hamamsy and colleagues (8). This approach optimizes valve coaptation, reduces the risk of autograft dilatation, and enhances long-term durability. In all cases, a meticulous intraoperative evaluation of the autograft was performed to confirm cusp symmetry and mobility, followed by careful resection of the infundibular muscle to tailor it to the native aortic annulus. In patients with dilated aortic annuli (≥27 mm) or predominant aortic insufficiency, an extra-anatomic annuloplasty using a polytetrafluoroethylene (PTFE) or Dacron ring was systematically performed to stabilize the neo-root and prevent late valvular insufficiency. In cases with dilatation of the sinotubular junction or ascending aorta (>40 mm), replacement of the ascending aorta with a Dacron graft was performed, which also reinforced the distal anastomosis of the autograft. For moderate dilatations (35–40 mm), a short-interposed graft was used to stabilize the sinotubular junction. These measures, forming part of our standardized technique, aim to preserve autograft geometry and function, limit progressive dilatation, and improve procedural durability as proposed by El-Hamamsy as well (7, 8). This combination—deep sub-annular implantation, external ring annuloplasty of the annulus, and supra-commissural Dacron replacement of the ascending aorta when indicated—is the standard operative template of the program. A full Dacron-encased inclusion strategy has not been adopted routinely at our institution.

Postoperative management includes an aggressive systolic blood pressure control strategy during the first year, maintaining maximum systolic pressures between 110 and 120 mmHg to minimize the risk of autograft dilatation. In addition, a low-dose nonsteroidal anti-inflammatory regimen (ibuprofen 200 mg every 12 h) is prescribed for six months to reduce the local inflammatory response against the homograft and promote long-term durability. Both were adopted following the Ross Masters Group “10 Commandments” consensus (7, 8) and institutional experience at high-volume Ross centers, where low-dose NSAID therapy has been proposed to attenuate the early inflammatory response directed against the cryopreserved pulmonary homograft, potentially mitigating accelerated homograft calcification. The dose is intentionally low and given with gastric protection; it is withheld in patients with renal dysfunction, active bleeding, or peptic ulcer disease, and is interrupted earlier if any intolerance arises. We acknowledge that direct randomized evidence for this strategy is still lacking and have included this point as a limitation.

## Results

Baseline clinical characteristics are summarized in Table 2. Overall, a total of 29 patients who underwent the Ross procedure were included. The median age was 47 years (range: 21–61). Four patients (13.8%) were female. The median EuroSCORE II score was 2.76% (range: 1.08–4.49). The median preoperative ejection fraction was 62% (40–80). The main surgical indications were aortic stenosis (34.5), aortic regurgitation (27.6%) and mixed lesions (34.5%). There was 1 endocarditis. Bicuspid aortic valve was present in 25 patients (86.2%), and two patients (6.9%) had unicuspid morphology. Patients exhibited a substantial comorbidity burden, with chronic heart failure and ascending aortic aneurysm being the most frequent conditions, followed by arterial hypertension and ascending aortic dilatation. Most patients were symptomatic at the time of surgery, predominantly in NYHA class II-III.

**Table 2:** Baseline clinical characteristics of 29 consecutive adult patients undergoing the Ross procedure.

Baseline characteristics	(*n*)	%
Age (years), median (range)	47 (21–61)	
Male/Female	25/4	86.2/13.8
EuroSCORE II (%), median (range)	2.76 (1.08–4.49)	
Left ventricular ejection fraction (%), median (range)	62 (40–80)	
Surgical indication		
Stenosis	10	34.5
Regurgitation	8	27.6
Mixed lesion	10	34.5
Endocarditis	1	3.4
Aortic valve morphology		
Unicuspid	2	6.9
Bicuspid	25	86.2
Tricuspid	2	6.9
NYHA functional class		
NYHA I	10	34.5
NYHA II	11	37.9
NYHA III	8	27.6

Values are expressed as *n* (%) or median (range). EuroSCORE II, European System for Cardiac Operative Risk Evaluation II. NYHA, New York Heart Association.

Detailed operative characteristics, including pulmonary homograft type, size, and concomitant procedures, are summarized in Table 3. The median aortic cross-clamp time was 205 min (range: 150–359), and the median cardiopulmonary bypass time was 230 min (range: 179–449). The median diameter of pulmonary homografts was 26 mm (range: 23–27). Stabilization techniques were applied in 25 patients (86.2%), most commonly ascending aortic replacement (*n* = 19) and aortic annuloplasty (*n* = 17). Additional procedures were performed in 7 patients (24.1%), including 2 septal myectomies for hypertrophic obstructive cardiomyopathy, 2 patent foramen ovale closures (one combined with tricuspid annuloplasty), one reimplantation of an anomalous right coronary artery, one right-sided MAZE with left atrial appendage closure, and two hemiarch replacement.

**Table 3:** Intraoperative data for 29 consecutive patients undergoing the Ross procedure. Values expressed as *n* (%) or median (range).

Intraoperative data	(n)	%
Cross-clamp time (min), median (range)	205 (150–359)	
Cardiopulmonary bypass time (min), median (range)	230 (179–449)	
Pulmonary homograft size (mm), median (range)	26 (23–27)	
Autograft stabilization techniques		
External aortic annuloplasty (PTFE or Dacron ring)	*n* = 17	58.6%
Ascending aortic replacement (Dacron tube)	*n* = 19	65.5%
Sinotubular junction stabilization graft	*n* = 3	10.3%
Additional procedures	*n* = 7	24.1%
Septal myectomy	*n* = 2	6.9%
Patent foramen ovale closure	*n* = 2	6.9%
Tricuspid annuloplasty	*n* = 1	3.4%
Reimplantation of an anomalous right coronary artery	*n* = 1	3.4%
Right-sided MAZE with left atrial appendage closure	*n* = 1	3.4%

PTFE, polytetrafluoroethylene; MAZE, Cox-maze ablation procedure.

Postoperative outcomes are summarized in Table 4. There were no operative deaths. One patient was on ECMO for post operative right ventricle failure and was successfully weaned at day 2. The median ICU stay was 3 days (range: 2–7), and the median total hospital stay was 6 days (range: 3–20). Perioperative stroke occurred in 3 patients (10.3%), as detailed in Table 5. All three events were ischemic in nature and of probable thromboembolic mechanism, favored by the combination of prolonged cardiopulmonary bypass times during the program's learning phase, and early atrial arrhythmia. Functional outcome at discharge was favorable in two patients (mRS: 0 and 1) and moderate in one (mRS: 3). All three lesions were predominantly cortical in the middle-cerebral-artery territory. No large-vessel occlusion requiring mechanical thrombectomy was identified, and the affected patients received neuroprotection, imaging surveillance, and therapeutic anticoagulation. Following these early events, the program introduced additional preventive measures for subsequent cases, including routine intraoperative epiaortic ultrasound scanning of the ascending aorta, a more liberal threshold for postoperative atrial fibrillation anticoagulation, and earlier reassessment of neurological status after extubation. Transient postoperative atrial fibrillation was observed in 4 patients (13.8%), and transient or definitive complete atrioventricular block in 2 patients (6.9%). Three permanent pacemakers were ultimately implanted (10.3%); in one patient, the indication had been established prior to the Ross procedure.

**Table 4:** Postoperative outcomes and of 29 consecutive patients. Values expressed as *n* (%) or median (range).

Postoperative outcomes and Follow-up	(n)	%
Operative mortality	*n* = 0	0%
ICU stay (days), median (range)	3 (2–7)	
Hospital stay (days), median (range)	6 (3–20)	
Perioperative Stroke	*n* = 3	10.3%
mRS 0–1 at discharge	*n* = 2	6.9%
mRS 3 at discharge	*n* = 1	3.4%
Pacemaker implantation	*n* = 3	10.3%
Postoperative atrial fibrillation	*n* = 4	13.8%
Reoperation for bleeding	*n* = 0	0%
ECMO	*n* = 1	3.4%
Follow-up		
All-cause mortality	*n* = 0	0%
Autograft or homograft reintervention	*n* = 1	3.4%
Alive at latest follow-up	*n* = 29	100%

Echocardiographic data are reported for patients with available data at each time point. Regurgitation grades classified per ASE guidelines (0 = none, 1 = trivial, 2 = mild, 3 = moderate, 4 = severe). No patient had severe autograft or homograft regurgitation at any time point. Operative mortality defined as 30-day mortality after Ross procedure. ICU, intensive care unit; mRS, modified Rankin Scale; ECMO, extracorporeal membrane oxygenation.

**Table 5:** Characterization of perioperative neurological events. All three events were ischemic strokes of probable embolic mechanism, occurring in the first 16 cases of the program.

	Patient 13 (Male, 54 years)	Patient 14 (Male, 56 years)	Patient 16 (Male, 52 years)
Indication	Aortic regurgitation	Aortic stenosis	Aortic stenosis
Valve morphology	Bicuspid	Bicuspid	Bicuspid
Concomitant procedure	Annuloplasty + Ascending aortic replacement	Anomalous RCA reimplantation	Ascending aortic replacement
Cross-clamp time (min)	178	249	174
Stroke type	Ischemic, embolic	Ischemic, embolic	Ischemic, embolic
Timing	3 days post-discharge	Postoperative day 0	Postoperative day 1
Postoperative atrial fibrillation	Yes	No	No
mRS at discharge	1	3	0

mRS, modified Rankin Scale; RCA, right coronary artery.

Median follow-up was 295 days (range: 30–662 days). One patient had to be reoperated at 9 months due to pulmonary homograft endocarditis. No deaths were recorded in the follow-up period.

**Figure 1:**
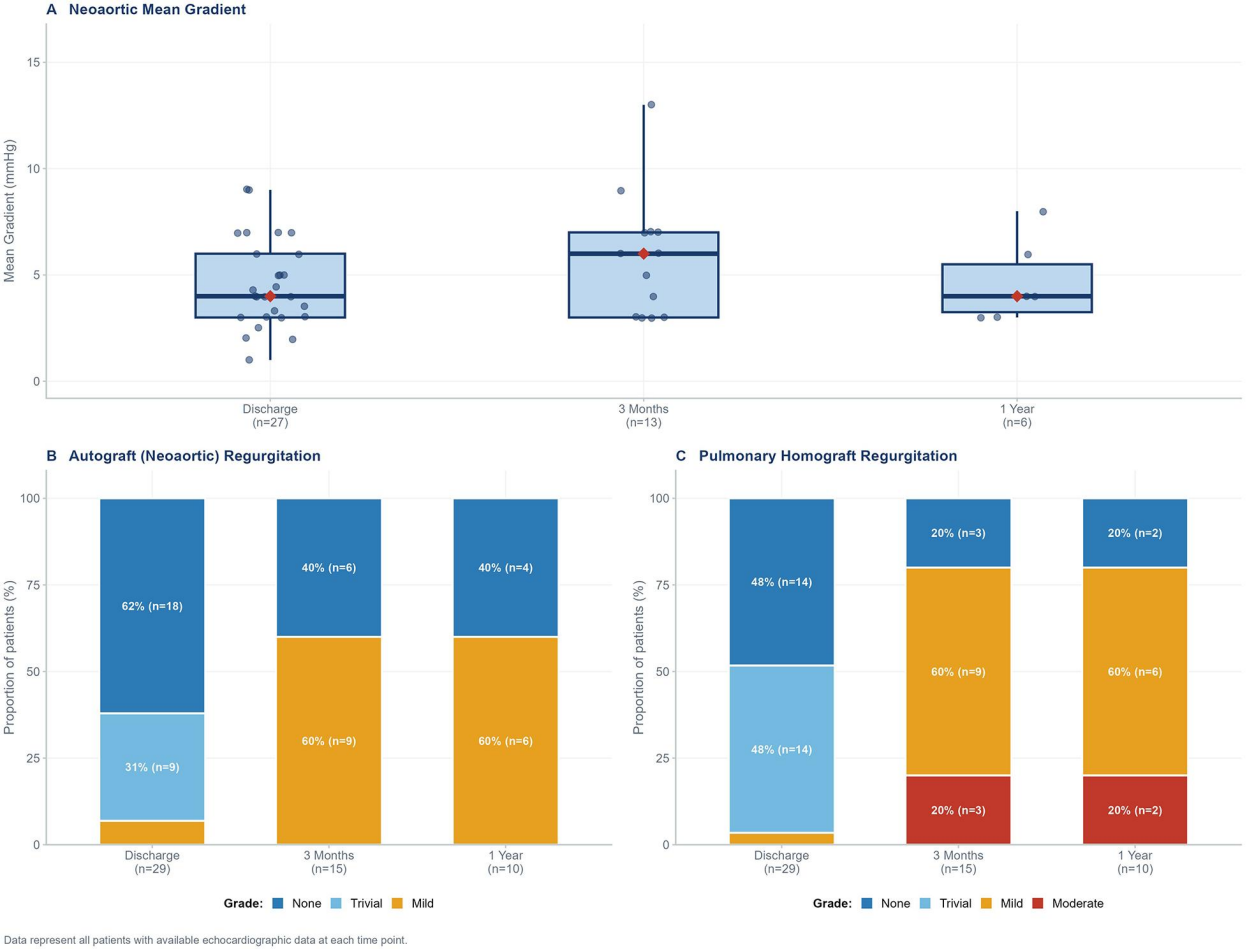
Serial echocardiographic outcomes following the Ross procedure. Serial transthoracic echocardiographic assessment in a consecutive, single-center cohort undergoing the Ross procedure. **(A)** Neo-aortic mean gradient (mmHg) at discharge, 3 months, and 1 year. **(B)** Distribution of neo-aortic (autograft) regurgitation severity over time. **(C)** Distribution of pulmonary homograft regurgitation severity over time.Regurgitation grades classified per ASE guidelines (0 = none, 1 = trivial, 2 = mild, 3 = moderate, 4 = severe); no cases of severe regurgitation were observed. Bar charts represent the proportion of patients at each grade, with corresponding absolute counts displayed. Sample sizes at each time point reflect available echocardiographic follow-up. LVEF, left ventricular ejection fraction; IQR, interquartile range.

Echocardiographic follow-up is summarized in Figure 1. At discharge, neoaortic regurgitation was absent in 18 patients (62%), trivial in 9 (31%), and mild in 2 (6.8%); no moderate or severe regurgitation was observed. Pulmonary homograft regurgitation was absent in 14 (48.3%) and trivial or mild in 15 (51.7%). The median neoaortic gradient was 4.0 mmHg (range: 1–9) and the median neopulmonary gradient was 2.0 mmHg (range: 1–5).

Three-month echocardiographic follow-up was available in 15 patients. Neoaortic regurgitation remained absent in 6 and mild in 9. Pulmonary homograft regurgitation was absent in 3 patients, mild in 9 and moderate in 3. No severe regurgitation was observed in any patient at any echocardiographic assessment.

One-year echocardiographic data were available in 10 patients from the first operative cohort. Autograft and homograft function remained preserved, with no severe regurgitation in any patient at one year.

## Comment

Over the last decade, multiple high-quality studies have shown several advantages of the Ross procedure that have led to a growing interest in its adoption by many cardiac surgery programs. A systematic review and meta-analysis including more than 3,500 adults demonstrated a 46% reduction in all-cause mortality and lower rates of stroke and major bleeding with the Ross procedure compared with mechanical aortic valve replacement (9). Population-based propensity-matched analyses from the United States and Canada have demonstrated that post-Ross survival not only exceeds that of both mechanical and bioprosthetic alternatives in young adults, but parallels that of the age- and sex-matched general population at 15 years of follow-up (4). A landmark propensity-matched cohort from the Toronto General Hospital confirmed lower all-cause mortality (HR: 0.35; 95% CI: 0.14–0.90; *p* = 0.028), lower rates of reintervention (HR: 0.21), valve deterioration (HR: 0.25), thromboembolic events (HR: 0.15), and permanent pacemaker implantation (HR: 0.22) following the Ross procedure compared with bioprosthetic aortic valve replacement in propensity-matched adults aged 16–60 years (10). Network meta-analytic data and single-center propensity-matched cohorts further confirm improved long-term survival, lower valve deterioration, and fewer valve-related complications when the Ross procedure is compared with bioprosthetic aortic valve replacement in young and middle-aged adults (11). The European Ross Registry encompassing 2,444 adult patients with a median follow-up of 17 years, demonstrates 25-year overall survival of 75.8% comparable to the general population, an autograft reintervention rate of 0.69% per patient-year, and freedom from RVOT reintervention of 0.62% per patient-year (12). Despite this growing body of evidence, current international guidelines still assign only a Class IIb recommendation to the Ross procedure in selected young patients, and it remains largely absent from routine decision algorithms for severe aortic stenosis (13). This disconnection between robust clinical evidence and limited adoption is driven, at least in part, by the technical complexity of the operation, concerns about long-term autograft and homograft durability and homograft availability (1, 2, 5).

In this initial series of 29 consecutive patients operated over approximately 22 months, the implementation of a Ross surgery program in a high-complexity university hospital in Latin America proved feasible and was associated with 0% operative mortality and only 1 reintervention due to endocarditis (no primary autograft failure). These findings are consistent with previously reported national and international series, supporting the Ross procedure as a valid option for young adults with aortic valve disease, while acknowledging that the limited cohort size and short follow-up time support only early safety and feasibility.

**Figure 2:**
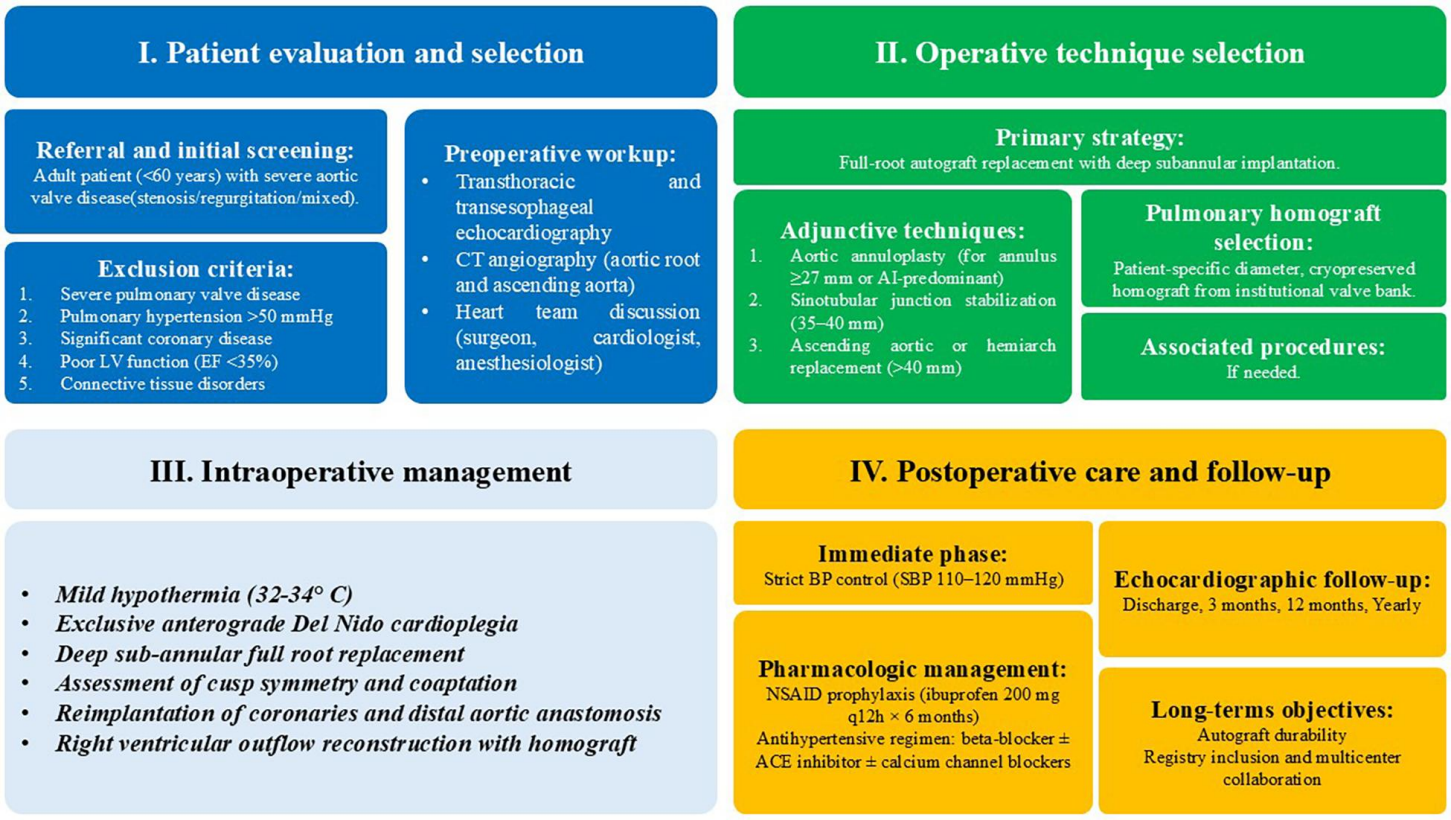
Institutional algorithm for patient selection, operative planning, and postoperative management within the Ross surgery program at the pontificia universidad católica de Chile.

Technical reviews and expert consensus emphasize that optimal outcomes rely on meticulous execution of key steps, such as deep sub-annular implantation, annular and sinotubular junction stabilization, and appropriate right ventricular outflow tract reconstruction, and advocate for concentrating the procedure in dedicated reference centers with multidisciplinary expertise (1, 5, 14). Institutional criteria for patient selection and technical considerations on operative planning are summarized in Table 1, and a visual algorithm summarizing selection and management is presented in Figure 2. The visual workflow and selection criteria may serve as a transferable template for centers initiating a Ross program, reflecting our experience with a heterogeneous population including aortic regurgitation, associated aortopathy, bicuspid and unicuspid morphology, and concomitant procedures. Making decision points explicit may facilitate benchmarking across institutions and future multicenter collaboration. The systematic use of deep sub-annular implantation with annular stabilization in patients with aortic regurgitation or annular dilatation, together with strict postoperative blood-pressure targets and prophylactic NSAID therapy, provides a reproducible pathway that can be audited and refined over time.

Unlike many published series that selectively include low-risk patients with isolated aortic stenosis, our cohort reflects the true complexity of aortic valve disease in a tertiary referral setting: 27.6% had predominant aortic regurgitation, 65.5% underwent concomitant ascending aortic replacement, and 24.1% required additional cardiac procedures. The acceptability of early safety outcomes in this heterogeneous, anatomically complex population supports the broader applicability of the Ross procedure when rigorous patient selection and systematic surgical planning are applied. This is consistent with the expanding indication philosophies advocated by contemporary expert centers and the “programmatic” model proposed in the surgical literature (1, 7).

From an organizational perspective, the program relies on a stable operative team of two attending surgeons with dedicated Ross training, a fixed anesthesia and perfusion team, and a nurse-led follow-up pathway, and on a prospective institutional registry in which every Ross case is captured at baseline, discharge, and at 3-month, 1-year, and annual echocardiographic time points. Of 158 adults who underwent aortic valve surgery during the study period, 29 (18.3%) were selected for the Ross operation, an uptake consistent with other programs in the implementation phase. Case volume evolved from 12 cases in the first 12 months to 17 cases in the subsequent 10 months, reflecting a progressive increase in institutional confidence. We observed a decrease in median cardiopulmonary bypass time between the first and second halves of the cohort (from 245 to 221 min) and clustering of the three strokes in the first half of the series, patterns compatible with a learning curve effect also described by Mazine and colleagues (10). These elements, together with the standardized operative template, written postoperative orders, and a minimum case-volume threshold of ten Ross operations per year per surgeon, form the basis of what we refer to as “programmatic implementation” and can be replicated by other high-complexity centers with a comparable structure. We recognize, however, that a full maturity assessment of the program will require longer follow-up and reassessment of these metrics in subsequent cohorts.

Table 6 summarizes prior Chilean experience, along with some of the main international series. Turner et al. reported an operative mortality of 2.3% in 131 patients who underwent the Ross procedure at the Instituto Nacional del Tórax between 1996 and 2006, one the first published Latin America series, with 10-year freedom from autograft reoperation of 93% (15). More recently, Torres-Herrera et al. updated this cohort to 161 patients operated between 1996 and 2012, showing freedom from reoperation of 83.2% and overall survival of 85.7% at nearly 20 years of follow-up (16). However, these cohorts reflect an earlier era, with heterogeneous techniques and limited description of standardized institutional pathways. Moreover, the Ross procedure remains infrequently performed in Chile and Latin America, and there is a paucity of contemporary data from university-based, high-complexity centers. Against this background of strong international evidence but low regional uptake, there is a clear need to evaluate the feasibility, safety, and early performance of structured Ross programs in our setting. Our contemporary cohort benefits from the understanding of autograft stabilization techniques, standardized postoperative management, prospective echocardiographic surveillance, and a dedicated multidisciplinary team, reflecting the evolution of programmatic Ross surgery over the intervening two decades. Although our series involves fewer patients and a much shorter follow-up, early safety outcomes are aligned with those reported in these national experiences.

**Table 6:** Comparison of the present series with selected published Ross procedure series.

Series	Type	N	Country	Period	Median Age	Op. Mortality	Stroke	Pacemaker	Follow-up	Freedom from Reoperation
Turner et al. (15)	SC	131	Chile	1996–2006	NR	2.3%	NR	0.7%	∼4.7 years	96% at 10 years
Torres-Herrera et al. (16)	SC	161	Chile	1996–2012	NR	NR	NR	NR	∼20 years	83.2% at ∼20 years
El-Hamamsy et al. (4)	PSM	434	USA	1997–2014	43 y	0.2%	2.4%	NR	12.5 years	NR
Mazine et al. (10)	PSM	108	Canada	1991–2018	41 y	0%	0.9%	1.9%	8.0 years	NR
Martin et al. (19)	SC	310	Canada	1990–2014	40.8 y	NR	NR	NR	∼12 years	76.1% at 20y
Notenboom et al. (20)	RCT	108	Canada	1995–2010	38 y	0.9%	NR	NR	24.1 years	80.3% autograft at 25 years
Yokoyama et al. (11)	NMA	NMA	Multi	Various	NR	0,5%	1,6%	NR	6.3 years	Superior vs mechanic/biologic valve
Aboud et al. (12)	Reg	2,444	Europe	1988–2018	38 y	1.0%	NR	NR	9.2 years	0.69%/patient year autograft
Flynn et al. (17)	MA	6,059	Multi	Various	NR	1.73%	1.5%	NR	6.0 years	76.1% at 20y
Present series	SC	29	Chile	2024–2026	47	0%	10.3%	10.3%	295 days	96.6% at 295 days

Values are reported as published in each source. Operative mortality is defined as 30-day or in-hospital mortality. Stroke rates are perioperative (30-day) unless otherwise specified. Survival and freedom from reintervention estimates are Kaplan–Meier actuarial estimates at the stated time intervals; see individual references for definitions. Where specific data were not reported in the source publication, NR (not reported) is indicated. SC, single-center observational; PSM, propensity score-matched analysis; Reg, multicenter registry; NMA, network meta-analysis; MA, systematic review and meta-analysis; RCT, randomized controlled trial follow-up; NR, not reported.

When placed alongside contemporary Ross series, our early operative times are comparable to those reported by Mazine et al. (median clamp: 146–180 min in high-volume reference centers) but remain somewhat longer, in keeping with the initial phase of a new program and the high proportion of concomitant aortic procedures.

The perioperative stroke rate of 10.3% (3 of 29 patients) represents the most prominent safety concern of this series and warrants detailed analysis within the context of the published literature. Contemporary high-volume Ross programs report perioperative neurological event rates between 1% and 5% (4, 9, 10), and the pooled stroke rate in the Flynn et al. meta-analysis was 1.5% across 3,019 patients during total follow-up (17). This difference is clinically meaningful and should be interpreted in the context of a small, heterogeneous cohort in which a single event accounts for more than three percentage points, longer bypass times during the program's learning curve, an older median age (47 years) with a higher prevalence of atherosclerotic risk factors than in most published series. Contributing case-level factors included extreme operative complexity: one patient required concomitant anomalous right coronary artery reimplantation with a cross-clamp time of 249 min, one patient developed concurrent postoperative atrial fibrillation (a recognized cardioembolic mechanism), and all three cases involved ascending aortic replacement with associated aortic manipulation. All three neurological events were ischemic consistent with cardioembolic etiology. One case occurred on postoperative day 0, another on day 1 and one appeared three days after hospital discharge, and none of them with hemorrhagic events. Two of three patients achieved mRS: 0–1 at discharge, and one was discharged with mRS: 3 (moderate disability, ambulatory without assistance) with subsequent neurological rehabilitation. No neurological events have occurred in the subsequent 13 patients (cases: 17–29), suggesting the impact that the learning curve might have. Our data, derived from a single-center initiating its adult Ross program, are consistent with broader published literature in identifying the early phase as the period of highest procedural risk (18). These findings underscore the importance of prospective neurological monitoring and systematic weaning protocols during the learning curve of newly established Ross programs, and support concentrating the procedure in centers with dedicated team infrastructure rather than diffusing it to low-volume settings. The implication for regional dissemination is clear: structured program establishment with explicit learning curve monitoring, systematic protocol refinement, and concentration of cases in dedicated centers is not merely recommended but essential for patient safety.

The pacemaker rate of 10.3% (*n* = 3 of 29) is higher than that reported in most contemporary series and merits contextual analysis. Two of those three implantations (6.9%) were for complete atrioventricular block (CAVB), and likely reflects the depth of sub-annular implantation of the autograft, a well-recognized trade-off between conduction-system preservation and long-term stability of the neo-root. The Toronto series reported a CAVB/PM rate of approximately 4.0% (10), and Flynn et al. pooled 4.0% (17). The two CAVB events occurred in the first 18 cases; systematic intraoperative monitoring of conduction intervals after autograft seating, implemented as a protocol refinement, has been adopted to reduce this risk in subsequent cases. The third pacemaker indication was not CAVB-related and was determined preoperatively.

Regarding early hemodynamic results (median neoaortic gradient 4.0 mmHg, no patient with moderate or severe regurgitation) these are comparable to those reported in reference centers. The role of autograft stabilization techniques, in particular external annular reinforcement and ascending aortic replacement, has been highlighted by several groups as a key determinant of neo-root geometry and freedom from late autograft dilatation (1, 5, 7), and this consensus guided the systematic adoption of these adjuncts in the present program.

Despite its potential advantages, the Ross procedure remains underused in Chile and Latin America, likely because of its technical demands, the need for a dedicated surgical team, and limited access to pulmonary homografts. The development of a cryopreserved homograft bank was an operationally critical prerequisite for program launch, and its establishment represents a transferable model for institutions across Latin America considering Ross program development. The bank enabled uninterrupted homograft availability throughout the study period, eliminating the logistical barrier most frequently cited as a reason for not adopting the Ross procedure in resource-limited settings. Regional tissue banking initiatives, potentially coordinated at the national level, represent an infrastructure investment that would substantially lower the barrier to Ross adoption across the continent.

This study has inherent limitations. The sample size (*n* = 29) and follow-up duration (median: 295 days) are insufficient to evaluate autograft and homograft durability, freedom from reoperation at clinically meaningful time horizons, or long-term survival, the primary endpoints by which the Ross procedure is ultimately assessed. Three-month echocardiographic data are available in only 15 patients, and one-year data in 10 patients, limiting the statistical power of serial analyses. The observational, single-center, non-comparative design precludes causal inference or comparison with alternative surgical strategies. The number of adverse events is insufficient for multivariable analysis, and all event-level conclusions should be interpreted as hypothesis-generating. Despite these limitations, the prospective design, complete follow-up within the available observation window, formal statistical analysis and transparent reporting of all complications, provides contemporary data from a high-complexity university hospital and documents the structured implementation of an institutional program.

In conclusion, the implementation of a structured Ross procedure program at our university center in Latin America proved feasible and was associated with acceptable early postoperative outcomes: zero operative mortality, satisfactory early hemodynamic valve performance, and no autograft reinterventions over a median follow-up of approximately ten months. These observations support feasibility and early safety in our setting; they do not establish, and should not be extrapolated to, long-term safety or durability. Standardization of the procedure and systematic follow-up are crucial to ensure the safety and durability of these results. Expansion of the cohort, prolongation of follow-up, and multicenter collaboration within a national or regional registry are the essential next steps toward establishing the role of the Ross procedure in Latin American cardiovascular surgical practice.

## Data Availability

The raw data supporting the conclusions of this article will be made available by the authors, without undue reservation.
